# Incidence and correlates of high blood pressure from childhood to adulthood: the Birth to Twenty study

**DOI:** 10.1097/HJH.0000000000003004

**Published:** 2021-09-01

**Authors:** Romain Meer, Daniel Boateng, Kerstin Klipstein-Grobusch, Shane A. Norris, Juliana Kagura

**Affiliations:** a Julius Global Health, Julius Center for Health Sciences and Primary Care, University Medical Center Utrecht, Utrecht University, Utrecht, the Netherlands; b Department of Epidemiology and Biostatistics, School of Public Health, Faculty of Health Sciences; c SAMRC Developmental Pathways for Health Research Unit, Department of Paediatrics, School of Clinical Medicine, University of the Witwatersrand, Johannesburg, South Africa; d Global Health Research Institute, School of Human Development and Health, University of Southampton, UK

**Keywords:** adolescents, blood pressure, children, incidence, low-income and middle-income countries, risk factors, sub-Saharan Africa

## Abstract

**Background:**

There is growing evidence from high-income countries suggesting that hypertension developed in childhood and adolescence persists into adulthood. The objective of this study was to investigate the incidence and risk factors of high blood pressure (BP) in urban black children.

**Methods:**

We used data from the Birth to Twenty (BT20+) cohort in Johannesburg, South Africa constituting of children born in 1990 and who had their growth, development and blood pressure measured at six follow-up periods over the course of 13 years. High BP was classified as at least 95th percentile for age, sex and height. Incidence rate of high BP was calculated using survival analysis and risk factors were determined by use of Cox proportional hazard regression.

**Results:**

Over a follow-up period of 13 years, the overall incidence rate of high BP was 57 cases per 1000 person-years (95% CI 53.2–61.1). Risk for incident high BP increased with rapid relative weight gain in early childhood (hazard ratio =1.11, 95% CI 1.00–1.22), mid-childhood (hazard ratio = 1.13, 95% CI 1.03–1.24) and adolescence (hazard ratio = 1.21, 95% CI 0.99–1.47). Maternal parity significantly increased the risk for incident high BP (hazard ratio = 1.08, 95% CI 1.01–1.15).

**Conclusion:**

Maternal parity and relative weight gain were determinants for incident high blood pressure in urban black South African children and adolescents. To reduce the high incidence and the disease burden of high BP, national programs should focus on promoting healthy lifestyle in early stages of life to prevent rapid weight gain and later cardiovascular disease risk. Further research is required to investigate whether incident high BP in childhood predict clinical outcomes in adulthood.

## INTRODUCTION

Hypertension is a prominent global health concern and a major risk factor for cardiovascular diseases [1,2]. Although once uncommon in the sub-Saharan African populations, the prevalence of hypertension has risen significantly in the last decades [3–5]. Growing evidence suggests that hypertension is developed in childhood and in adolescence and persists into adulthood [6–11]. A large systematic review on the global prevalence of childhood hypertension showed that the prevalence in sub-Saharan African countries is nearly twice as large as the global prevalence (6.9 vs. 4%) [12]. In addition, childhood hypertension is approximately 25% more prevalent in low-income and middle-income countries (LMICs) than in high-income countries (HICs) (4.4 vs. 3.5%) [12]. These differences in prevalence can possibly be attributed to ethnic discrepancies. Modesti *et al.*[13] stated that the interaction of genes in sub-Saharan African individuals with the socioeconomic environment predispose them to relatively high risk of hypertension as compared with people from European decent. Moreover, the increasing prevalence of childhood hypertension in sub-Saharan Africa (SSA) is aggravated by the rapid urbanization and the epidemiological transition from a traditional lifestyle towards a western lifestyle [2,14].

Most of the studies regarding childhood hypertension and blood pressure (BP) tracking were conducted in HICs. The study by Kagura *et al.* represents one of the few studies that were conducted in LMICs [15]. This research group reported a high point prevalence of high-normal BP (9.2–16.4%) and high BP (8.4–24.4%) among South African children between 5 and 18 years of age. In addition, they reported a positive weak-to-moderate tracking of blood pressure. These findings suggest that children and adolescents with elevated BP should be identified in a timely manner in order to prevent morbidity and mortality from cardiovascular disease and other hypertension-associated diseases in adulthood.

There is need to describe the epidemiology of raised BP including risk factors in the paediatric populations in LMICs to inform timely interventions. The identification of risk factors underlying high BP is of paramount interest, as risk management may turn out to be more cost-effective in reducing the disease burden than treatment of hypertension [16].

The objectives of this study were to investigate new cases of high BP that emerge from early childhood to adulthood and identify risk factors that predict the incidence of high BP from early childhood in urban black South African children.

## METHODS

### Study sample

**FIGURE 1:**
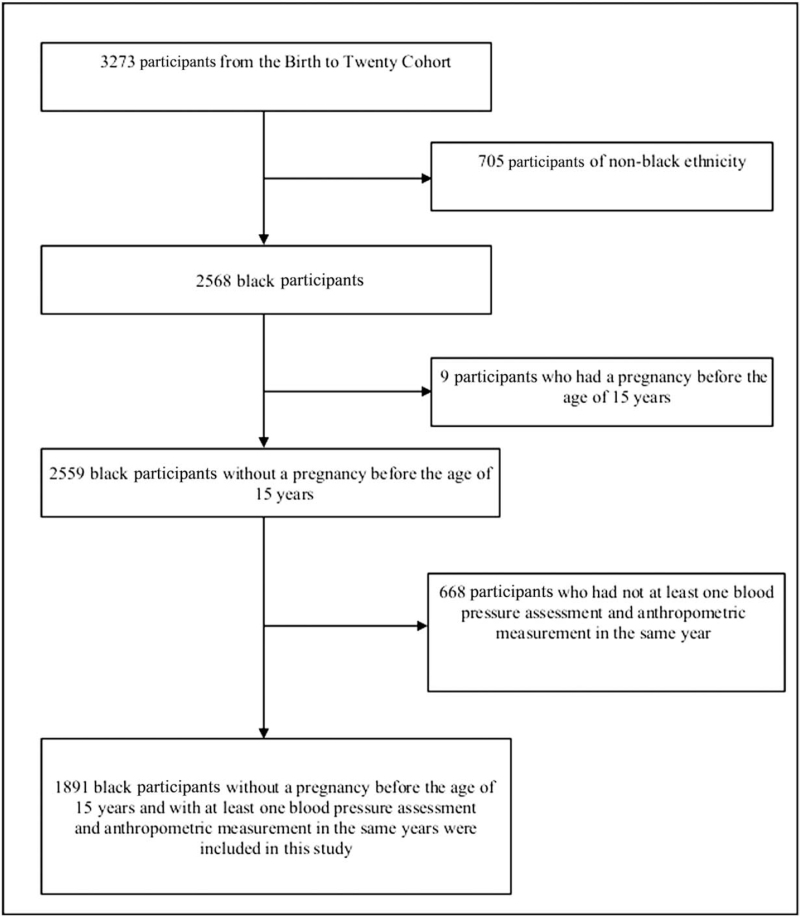
Study inclusion and exclusion criteria.

We used data from the Birth to Twenty (BT20+) cohort in Johannesburg, South Africa constituting of children born in 1990 and who had their growth and development as well as socioeconomic status (SES) assessed at several time points from childhood to adulthood. The cohort consisted of 3273 singleton infants who were born in the Soweto-Johannesburg Metropolis, South Africa. Pregnant residents who delivered their babies between 23 April 1990 and 8 June 1990, were recruited from antenatal clinics into the BT20+ cohort. Follow-up was conducted via telephone or field visits and contact between the researchers and participants/parents/caregivers was maintained by birthday cards or by newsletters. Further details of the recruitment and cohort attrition have been described by Richter *et al.*[17]. Only black participants who had not been pregnant before the age of 15 years and who had both blood pressure assessments and anthropometric measurements in one or more data waves (1995, 1998, 2002, 2003, 2005 and/or 2008) were selected for this study. Out of the 3273 participants from the BT20+ cohort, 705 participants (21.5%) were of nonblack ethnicity and were excluded from this study. Furthermore, nine black female individuals (0.3%) were also excluded as they had a pregnancy before the age of 15 years. An additional 668 black participants (20.4%) were excluded from the analysis as they did not have BP assessments for several reasons despite been seen and having anthropometric measurement within the same data wave. Blood pressure and anthropometric measurements of the children were taken on the same visit. But if the BP measurement could not be taken despite the weight and height, that point is excluded from the analysis. To summarize, 1891 participants (57.8%) (913 male participants; 978 female participants) in the BT20+ cohort were found to be eligible for this study. Each data wave consisted of the following number of participants: 1995 (*n* = 1021; 49% male participants), 1998 (*n* = 1022; 49% male participants), 2002 (*n* = 1240; 48% male participants), 2003 (*n* = 1383; 48% male participants), 2005 (*n* = 1617; 48% male participants) and 2008 (*n* = 1581; 48% male participants). Figure 1 shows the flow chart of study inclusion and exclusion.

### Measures

#### Anthropometry

The anthropometric measures were assessed by trained research assistants. Participants were measured while wearing light clothes and barefoot. The weight of the participant was measured using a digital scale and was measured to the nearest 0.1 kg. The participant's length was measured using a wall-mounted stadiometer (Holtain, Crymych, Pembrokeshire, UK) and was measured to the nearest 0.1 cm. BMI was calculated by dividing the weight (kg) by the height in square meters (m^2^). Furthermore, weight, height and BMI were converted to their respective WHO *z* scores stratified by age. Stunting was defined as height-for-age *z* score less than −2 [18]. The BMI *z* scores were used to compute latent classes (trajectories) constituting of participants that had similar levels and tempo of BMI development between ages 5 and 18 years. The trajectories were denoted as: 1 (normal weight), 2 (late onset overweight), 3 (early onset obesity to overweight) and 4 (early onset obesity to morbidly obese). The details of the computation of the BMI trajectories are described elsewhere [19].

#### Blood pressure

SBP and DBP were measured by trained research assistants. The BP of a 5-year-old participant was measured using a Dinamap Signs Monitor 1846SX (Critikon, Chicago, Illinois, USA). The BP of participants ranging from 8 to 18 years of age was measured using an Omron 6 automated machine (Kyoto, Japan). The blood pressure measurements were conducted in a seated position with the use of an appropriate cuff size. Three individual measurements were taken at a 2 min interval. Age-standardized, sex-standardized and height-standardized percentile tables for blood pressure classification in children and adolescents were used to classify the participant as either normotensive (<90th percentile), prehypertensive (≥90th and <95th percentile, or >120/80 mmHg if <90th percentile) or hypertensive (≥95th percentile) according to the fourth report of the National High Blood Pressure Education Program (NHBPEP) [20]. For this current study, as BP measurements were based on one occasion per data collection wave, participants in the at least 95th percentile for BP, age, height and sex were classified as having high BP.

#### Growth

The participant's birth weight and gestational age were obtained from birth notifications. Attributable to the high correlation of the participant's weight and height in longitudinal data, conditional growth variables were computed. Standardized residuals derived from sex-specific linear regressions were used to compute the conditional weight independent of height (relative weight gain) and conditional height independent of weight (relative height gain) [21,22].

#### Covariates

Characteristics of the participant and the mother were collected using standard questionnaires. The parents or the caregivers reported a count of household assets, which was then summed up to give a measure of SES score of the participant. The mother also reported how many children she had borne before the birth of the participant. This information was needed to quantify the parity and to identify the participant's mother as being primiparous or multiparous. Lastly, the mother provided information on whether she had breastfed the participant.

### Statistical analyses

For the descriptive analyses, *t* tests were used to assess significant differences in continuous variables between two groups whereas *χ*^2^ test were used for significant differences in binomial/categorical variables between two groups. Repeated measurement ANOVA tests were used to assess significant differences in continuous variables between different data waves.

Disease incidence rate was calculated by dividing the number of new cases of high BP over the study period by the total person-years at risk using staggered entry survival analysis [23]. This method is a modified Kaplan–Meier approach that calculates the disease incidence while allowing gradual entry into the survival analyses. Although all participants of the BT20+ cohort were recruited at birth in 1990, many participants did not have their anthropometric measurement and BP assessment at age 5 years when we first collected BP data. However, majority of these participants were seen at a subsequent data collection wave. As such, these participants enter the survival analyses at a later data wave. Time to event is defined as the time between the first complete assessment and the first reported reading of blood pressure.

Participants who experienced the event were censored immediately after this time point. Participants who remained normotensive (normal) or prehypertensive (high-normal) up to the last year of follow-up were kept in the analysis after which they were censored. Participants with interval-censored data were assumed to be free of hypertension during this interval. A log-rank test was used to assess equality of survivor functions between different strata and the model was checked whether it satisfies the assumption of proportionality.

Univariable Cox proportional hazard regressions were used to identify potential predictors of raised BP. Multivariable Cox proportional hazard regressions using backwards procedure were used to estimate the multivariable adjusted hazard ratios for incident high BP associated with risk factors at baseline. Covariates were included in the multivariable model if the corresponding *P* value was equal to or less than 0.25. In the first multivariable model, all variables with a *P* value less than 0.25 in the univariable regression were included. Subsequently, the variable with the largest *P* value was step-wisely eliminated until all variables had a *P* value less than 0.1. Sex, maternal education and SES were, however, kept in the final model. We checked for interaction and confounding effects between variables by adjusting for every two-way combination of input variable. In case of nonproportionality, Cox proportional hazard regression models with time-dependent variables were used to identify factors independently associated with high BP. We checked for multicollinearity of the final model by computing correlation matrix estimates of all included variables. Finally, we used Martingale residuals and Cox–Snell residuals to assess the goodness-of-fit of the final model. We did not adjust for the competing risk of death, as the number of deaths before the age of 18 was negligible (less than 10 deaths in the whole BT20+ cohort of 3273 subjects) and data on the dates of deaths were unavailable.

We used a probability value of 0.05 as cut-off point to assess statistical significance, unless stated otherwise. The descriptive analyses were carried out using SPSS software Version 25.0.0 [24], whereas the survival analyses were carried out using STATA software Version 16.1 [25].

### Data management and security

The parents/caregivers provided informed consent when the participant was a minor at each data wave for the cohort. Otherwise, the participant provided informed consent. Ethical approval for this study was granted from the Human Research Ethics Committee of the University of the Witwatersrand, Johannesburg (M130556). The participants were anonymized in the study by using unique identification numbers. There was no information relating to the identification of the participant.

## RESULTS

### Child and maternal characteristics

Table 1 shows the child and maternal characteristics of the included participants of which 51.7% were females. On average, maternal parity was 2.2. The majority of the participant's mothers had no formal education (58.2%). In addition, 22.3% of the participants and 6.3% of the participants had a stunted growth in early childhood (0–2 years) and mid-childhood (2–5 years), respectively.

**TABLE 1:** Study characteristics of black South African children in the Birth to Twenty study

Variables	*N* (%) (*n* = 1891)
Male sex	913 (48.3)
Gestational age (weeks), mean (SD)	37.9 (1.8)
Small for gestational age	235 (12.7)
Birth weight (g) [*n* (%)]	3072 (502)
Low birth weight (<2500 g), mean (SD)	204 (10.8%)
Stunted growth	
Early childhood	223 (22.3)
Midchildhood	77 (6.3)
Socioeconomic status score	3.4 (1.6)
Maternal age (years)	25.7 (6.2)
Maternal education	
No formal education	1024 (58.2)
Up to secondary	592 (33.6)
Postsecondary education	144 (8.2)
Maternal parity	2.2 (1.3)
Mother ever breastfed the participant	1775 (95.4)
BMI trajectories	
Normal weight	1571 (86.1)
Late onset overweight	157 (8.6)
Early onset obese to overweight	54 (3.0)
Early onset obese to morbidly obese	42 (2.3)

SD, standard deviation.

The included and excluded participants were compared with respect to child characteristics and maternal characteristics to assess whether missingness was random (Supplementary Table 1). The included subjects had a significantly lower gestational age and a lower SES score as compared with the excluded participants. A significantly lower fraction of the included participants was classified as being small for their gestational age while a significantly higher fraction of the included participants had a stunted growth in early childhood. In addition, the mothers of the included participants were overall younger at the moment of birth of the participants, had less children and were more likely to breastfeed. Maternal education was significantly different between these two groups.

Results of the anthropometric measurements, BP assessments and proportions of blood pressure status between male and female participants is presented in Supplementary Table 2. Overall, male participants had a significantly lower weight, BMI and DBP as compared with female participants. However, male participants had a significantly higher SBP than female participants. The average age, height and high BP status did not differ significantly between male and female pargticipants.

We also compared participants who developed high BP somewhere during the study period with participants who remained normotensive during the entire study period (Table 2). Of the 1891 participants, 794 participants (42%) were identified as high BP cases somewhere between 1995 and 2008. In total, 197 participants (10.4%) were lost to follow-up and 900 participants (47.6%) who had their last follow-up in 2008 were normotensive. A significantly larger proportion of the raised BP participants were small for their gestational age as compared with their normotensive peers. In addition, mothers of high BP participants had significantly more children than mothers of normotensive participants. Participants with raised BP experienced a significantly higher relative height gain in mid-childhood as compared with normotensive participants. Finally, participants with high BP experienced a significantly higher relative weight gain in early childhood, in mid-childhood and in adolescence as compared with normotensive participants.

**TABLE 2:** Child and maternal characteristics of normotensive participants and high blood pressure participants

Child and maternal characteristics	Normotensive (*n* = 1097)	High blood pressure (*n* = 794)	*P* value
Male sex	526 (47.9)	387 (48.7%)	0.734
Gestational age (weeks), mean (SD)	37.9 (1.8)	38.0 (1.8)	0.145
Small for gestational age	118 (11.0)	117 (14.9)	0.012
Birth weight (g), mean (SD)	3078 (492)	3063 (516)	0.525
Low birth weight (<2500 g)	124 (11.3)	80 (10.1)	0.393
Stunted growth			
Early childhood	124 (22.2)	99 (22.6)	0.890
Mid-childhood	48 (7.1)	29 (5.3)	0.196
Socioeconomic status score	3.4 (1.6)	3.4 (1.7)	0.923
Maternal age (years)	25.7 6.2)	25.8 (6.2)	0.651
Maternal education:			0.530
No formal education	584 (57.1)	440 (59.6)	
Up to secondary	350 (34.2)	242 (32.8)	
Postsecondary education	88 (8.6)	56 (7.6)	
Maternal parity	2.1 (1.3)	2.3 (1.4)	0.010
Mother ever breastfed the participant	1032 (95.6)	743 (95.1)	0.669
Relative height gain			
Early childhood	−0.069 (0.939)	−0.058 (0.980)	0.839
Mid-childhood	−0.067 (1.026)	0.071 (1.039)	0.027
Adolescence	0.014 (0.966)	−0.046 (0.985)	0.337
Relative weight gain			
Early childhood	0.033 (0.998)	0.166 (0.943)	0.018
Mid-childhood	−0.128 (1.035)	0.034 (1.049)	0.010
Adolescence	−0.072 (0.906)	0.131 (1.036)	0.001
BMI trajectories			0.032
Early onset obese to overweight	890 (88.0)	651 (83.9)	
Normal weight	83 (8.0)	72 (9.3)	
Late onset overweight	24 (2.3)	29 (3.7)	
Early onset obese to morbidly obese	17 (1.6)	24 (3.1)	

SD, standard deviation.

### Survival analyses

**FIGURE 2:**
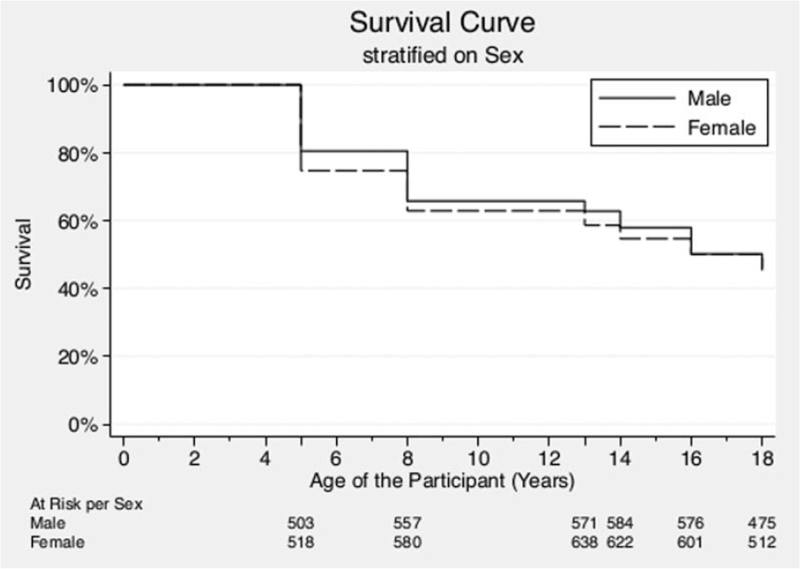
Sex-stratified survival curve with the numbers at risk per mean age of the participant at every data.

After accounting for staggered entry and lost to follow-up, overall probability of survival was 45.7% (95% CI 43.2–48.2). Whenever stratified by sex, the survival probabilities were 46.1% (95% CI 42.5–49.6) and 45.3% (95% CI 41.8–48.8) for male and female participants, respectively (Fig. 2). The survival probability did not differ significantly between male and female participants. The incidence rate of high BP in this sample is presented in Table 3. The overall hypertension incidence rate was 57 cases per 1000 person-years (95% CI 53.2–61.1). The incidence rate of high BP was 57.1 cases per 1000 person-years (95% CI 51.7–63.1) and 56.9 cases per 1000 person-years (95% CI 51.7–62.7) for male and female participants, respectively. The incidence rate of children of multiparous mothers was significantly higher than of children who were an only-child at the moment of birth. Moreover, the incidence rate of high BP of children who are small for their gestational age is significantly higher than of children who have a normal or large size for their gestational age. The sex-stratified incidence rate of raised BP per data wave is shown in Supplementary Table 3. The overall incidence rate did not differ significantly between male and female participants.

**TABLE 3:** Report of cases with number of person-years, incidence rate per 1000 person-years and the 95% confidence interval for each binomial and categorical explanatory variable

Variable	High blood pressure cases	Person-years	Incidence rate per 1000	95% CI			*P* value
	(*n*)	(%)		Person-years	Lower	Upper	
Sex	794		13 929				0.971
Male	387	48.7	6780	57.1	51.7	63.1	
Female	407	51.3	7149	56.9	51.7	62.7	
Maternal education	794		13 929				
No formal education	440	55.4	7508	58.6	53.4	64.3	
Up to secondary	242	30.5	4534	53.4	47.1	60.5	0.667
Postsecondary	56	7.1	986	56.8	43.7	73.8	0.834
Missing	56	7.1	901				
Maternal parity	794		13 929				0.014
Only child	289	36.4	5662	51.0	45.5	57.3	
Multiparous	505	63.6	8267	61.1	56.0	66.7	
Maternal age (years)			13 923				
≤24	371	46.7	6716	55.2	49.9	61.2	
25–34	335	42.2	5942	56.4	50.7	62.8	0.7913
≥35	88	11.1	1265	69.6	58.4	85.7	0.055
Birth weight less than 2500 g	794	100.0	13 929				0.247
No	713	89.8	12 314	57.9	53.8	62.3	
Yes	80	10.1	1583	50.5	40.6	62.9	
Missing	1	0.1	32				
Mother ever breastfed the participant	794		13 929				0.805
No	38	4.8	646	58.8	42.8	80.8	
Yes	743	93.6	3109	56.7	52.7	60.9	
Missing	13	1.6	174				
Small for gestational age	794		13 929				0.048
No	667	84.0	11 951	55.8	51.7	60.2	
Yes	117	14.7	1713	68.3	57.0	81.9	
Missing	10	1.3	265				
Stunted growth early childhood	794		13 929				0.448
No	340	42.8	6398	53.1	47.8	59.1	
Yes	99	12.5	1709	57.9	47.6	70.5	
Missing	355	44.7	5822				
Stunted growth mid-childhood	794		13 929				0.192
No	517	65.1	9763	53.0	48.6	57.7	
Yes	29	3.7	700	41.4	28.8	59.6	
Missing	248	31.2	3466				
BMI trajectories	776	100.0	13 848				
Normal weight	651	83.9	12 025	54.1	50.1	58.5	
Late onset overweight	72	9.3	1232	58.4	46.4	73.6	0.813
Early onset obese to overweight	29	3.7	364	79.7	55.4	114.6	0.079
Early onset obese to morbidly obese	24	3.1	227	105.7	70.9	157.7	0.0052

*P* values of maternal education category are in vertical order ‘No Formal Education vs. Up to Secondary’, ‘Up to Secondary vs. Post-Secondary’ and ‘Post-Secondary vs. No Formal Education’, respectively. CI, confidence interval.

#### Cox proportional hazard regressions

Results of the univariate Cox proportional hazard regressions are shown in Supplementary Table 4. Having a multiparous mother was significantly associated with the incidence of raised BP (hazard ratio = 1.18, 95% CI 1.02–1.37). Similarly, an increase of one in maternal parity significantly increased the risk of the participant to develop high BP by 8% (hazard ratio = 1.08, 95% CI 1.02–1.13). Being small for your gestational age also increased the risk of developing high BP (hazard ratio = 1.20, 95% CI 0.99–1.46). Lastly, increased relative weight gain in early childhood (hazard ratio = 1.11, 95% CI 1.02–1.21), in mid-childhood (hazard ratio = 1.12, 95% CI 1.03–1.21) and in adolescence (hazard ratio = 1.14, 95% CI 1.04–1.26) were associated with the risk of developing raised BP. Every variable, except ‘relative weight gain during adolescence’, met the proportional hazard assumption. A univariate Cox proportional hazard regression with a time-dependent variable was computed. This variable remained a borderline significant predictor for the development of high BP (hazard ratio = 1.21, 95% CI 1.00–1.46).

In multivariable analysis (Table 4), the risk of developing high BP increased significantly with maternal parity. An increase in maternal parity by one significantly increased the risk for incident high BP by 8% (hazard ratio = 1.07, 95% CI 1.00–1.15). Furthermore, the risk for incident high BP increased with a rapid relative weight gain in early childhood (hazard ratio = 1.10, 95% CI 1.00–1.22), in mid-childhood (hazard ratio = 1.16, 95% CI 1.06–1.28) in adolescence (hazard ratio = 1.13, 95% CI 1.00–1.27) early onset of obesity to overweight (hazard ratio = 1.72, 95% CI 1.04–2.85). There were no significant interactions or confounding effects between any of these four variables. Further, no multicollinearity was observed between any of the explanatory. We also assessed the interaction between small for gestational age (SGA) and subsequent weight gain but found no significant interaction between them [SGA × relative weight gain early childhood (*P* = 0.080); SGA × relative weight gain mid-childhood (*P* = 0.389); SGA × relative weight gain early adolescence (*P* = 0.635)].

**TABLE 4:** Multivariable Cox proportional regression analyses of incident high blood pressure and its correlates from childhood to adulthood

Variable	Adjusted hazard ratio	95% CI		*P* value
		Lower	Upper	
Maternal parity (no units)	1.07	1.00	1.15	0.065
Relative weight gain early childhood (no units)	1.10	1.00	1.22	0.069
Relative weight gain mid-childhood (no units)	1.16	1.06	1.28	0.002
Relative weight gain adolescence (no units) × ln (time)	1.13	1.00	1.27	0.045
BMI trajectories	1.00			
Normal weight (ref)	0.82	0.57	1.18	0.291
Late onset overweight	1.72	1.04	2.85	0.034
Early onset obese to overweight	1.49	0.80	2.78	0.204
Early onset obese to morbidly obese				
Socioeconomic status	0.98	0.92	1.05	0.615
Maternal education (Ref = lower)	1.11	0.89	1.39	0.360
No education (ref)	1.12	0.77	1.64	0.556
Up to secondary				
Postsecondary				
Female sex	1.08	0.88	1.32	0.477

The variable ‘relative weight gain adolescence’ violated the proportional hazard assumption. This variable was made time-dependent. Model adjusted for the variables shown. CI, confidence interval. Psuedo *R*^2^ = 0.044.

To examine whether there were sex differences in risk factors, we stratified the multivariable analysis for males and female participants (Supplementary Table 5). For male participants, relative weight gain in early childhood (hazard ratio = 1.13, 95% CI 0.99–1.30), in mid-childhood (hazard ratio = 1.16, 95% CI 1.02–1.33) and in adolescence (hazard ratio = 1.38, 95% CI 1.05–1.82) were associated with incident-raised BP. For female participants, maternal parity remained a significant predictor of incident high BP (hazard ratio = 1.49, 95% CI 1.14–1.96).

## DISCUSSION

Our study reports a high incidence of high BP in South African children and adolescents. In addition, we identified that a high maternal parity and a rapid relative weight gain in early childhood, in mid-childhood and in adolescence are associated with an increased risk of hypertension.

We reported an overall incidence of hypertension of 57 cases per 1000 person-years. Given the scarcity of studies reporting the incidence rate of hypertension among African children [26], comparison with other studies undertaken in SSA is very limited. Goon *et al.* estimated the incidence of hypertension among 296 rural South African children aged 7–13 years and reported an incidence rate of 0.0–0.4% [27]. The large difference in incidence rate in the study by Goon *et al.*, may be explained by the considerably lower sample size with a maximum of 63 children per data wave and the rural study setting. A landmark study by Seedat and colleagues from 1982 showed that BP levels and the prevalence of hypertension in rural populations are lower than in urban populations [28]. As observed by Modesti *et al.,* rising incidence of hypertension and other cardiovascular diseases is associated with urbanization and related epidemiological transition, which is more apparent in urban areas as compared to rural areas [13].

Our findings suggest that children and adolescents have an increased risk of incident hypertension as the maternal parity increases. However, Gaillard *et al.*[29] examining associations between maternal parity and childhood growth characteristics in mainly European women and their children observed no association between maternal parity and SBP and DBP. We speculate that our finding could be a result of hidden maternal characteristics associated with maternal parity, such as maternal blood pressure or the presence of preeclampsia [30–32]. Unfortunately, we did not include such variables in our analyses as this data was not collected in the cohort study, and thus we were not able to investigate whether there was a confounding effect of any of these variables on the association between maternal parity and incident hypertension.

Maternal health factors, such as anaemia can also impact fetal development and possibly subsequent risk for hypertension. Previous systematic reviews and meta-analyses, involving 68 articles [33] and 26 articles [34] found a positive association between maternal anemia and low birth weight in LMICs. In South Africa, there are only a few studies and the evidence is inconclusive as Symington *et al.* in 2019 showed a positive association between maternal anemia and LBW [35] whereas Tshotetsi in 2019 reported no association [36].

The findings of this study further suggest that a relative weight gain in early childhood, in mid-childhood and in adolescence increases the risk of developing high BP. This finding corroborates that of a study by Adair *et al.*[21], which used data from five prospective birth cohorts from LMICs, including this current cohort. They reported that relative weight gain in early childhood, in mid-childhood and in adolescence were positively associated with having a high BP. In addition, the study by Adair *et al.* showed that this association was stronger for male participants as compared with female participants. This finding is in line with our study, as the sex-stratified multivariable analysis showed that the hazard ratios for relative weight gain in mid-childhood and in adolescence are higher in male participants than in female participants. Moreover, in our study, relative weight gain in female participants was no longer a significant predictor of incident high BP, implying that relative weight gain is more problematic in male participants. However, the study of Adair showed that relative weight gain in female participants is a significant predictor of hypertension although the direction of association was the same. The finding in the study by Adair may be because of a small sample size for the sex-stratified analyses for the South African cohort.

The high incidence of high BP and the relative weight gain as risk factor suggest that preventive measures focussing on lifestyle modification should be taken to reduce the disease burden of hypertension. Focusing on preventing relative weight gain by promoting a healthier lifestyle through regular physical activity and healthy nutrition in early life should be one of the primary concerns in improving cardiovascular health.

### Strengths and limitations

One of the strong aspects of this study was the high number of participants included in this study, low number of lost to follow-up and the long duration of follow-up. No significant differences in characteristics of participants who were lost to follow-up and the rest of the included participants, were observed enhancing the generalizability of the findings. Another strength of this study was the use of the conditional growth variables – relative height gain and relative weight gain – to deal with multicollinearity of growth variables. Blood pressure data was obtained by using automated BP monitors leading to a reduced interobserver variation in BP measurements and made the measured SBP and DBP variables more robust. The BP of a 5year-old participant and those from 8 to 18 years was measured using a Dinamap Signs Monitor 1846SX (Critikon) and an Omron 6 automated machine, respectively. In a previous evaluation of BP monitors for use in children, BP values obtained using the Dinamap hardly compared with those obtained with Omron [37]. However, of the two, the Dinamap has been suggested to have generally less accurate measurements of BP [38]. Other evidence, however, suggests that the Dinamap is a more appropriate instrument of choice in children [37] whereas the Omron device could be reliable and valid for adolescents [39].

Finally, BP status was assessed by using a classification system, which incorporates sex, age and height. These three variables are the main confounders for hypertension status in growing individuals. Blood pressure standards based on these variables provide a more precise classification of hypertension status according to body size and avoids misclassification of children who were very short or very tall [20].

We cannot rule out white-coat effect among children, which could overestimate the incident hypertension [40,41]. This could possibly explain high BP measurement taken at 5 and 8 years, whereas lower values are recorded thereafter. However, almost 36% of the children who had high BP status at age 5 years maintained that status at age 18 years, and there was 60% increased likelihood of maintaining BP status [15].

Shortcomings of the current study included differences in characteristics between included and excluded study BT20 participants for the current analysis with respect to some child and maternal characteristics, such as SES score, gestational age and maternal education. As we only included black participants in the analysis, results of this study may not apply to the general South African population. Furthermore, we assumed that participants who had interval-censored data were normotensive during this interval.

In conclusion, we have shown that the incidence of high BP in the current study is high and maternal parity and relative weight gain were observed to be risk factors for incident-raised BP in urban black South African children and adolescents. In order to reduce the high incidence and the disease burden of hypertension, national health programs should focus on promoting healthy lifestyle in early stages of life to curb rapid relative weight gain and prevent later cardiovascular disease risk. Further research is required to investigate whether incident high BP in childhood predict clinical outcomes in adulthood.

## ACKNOWLEDGEMENTS

Birth to Twenty was funded by the Wellcome Trust (UK), SA Medical Research Council and the DSI-NRF Centre of Excellence in Human Development at the University of the Witwatersrand, Johannesburg, South Africa.

### Conflicts of interest

There are no conflicts of interest.

## Supplementary Material

Supplemental Digital Content
