# Supplementary material for: Incidence and correlates of high blood pressure from childhood to adulthood: the Birth to Twenty study
Source: J Hypertens. 2021 Sep 1;40(2):274–82. doi: 10.1097/HJH.0000000000003004 (PMC8728753; doi:10.1097/HJH.0000000000003004)
Supplement: Supplemental Digital Content [file jhype-40-274-s001.docx]

**Supplementary Table 1. Child and maternal characteristics of included and excluded participants. Included**

**Value in brackets is the standard deviation or valid percentage of the group.**

| **Child & Maternal**  **Characteristics** | **Included**  **(n = 1891)** | **Excluded**  **(n = 1382)** | **p-value** | **Lost to Follow Up**  **(n = 197)** | **Not Lost to Follow Up**  **(n = 1694)** | **p-value** |
| --- | --- | --- | --- | --- | --- | --- |
| Male Sex | 913 (48.3) | 678 (49.1) | 0.660 | 90 (45.7) | 823 (48.6) | 0.441 |
| Gestational Age (weeks), mean (SD) | 37.9 (1.8) | 38.5 (2.0) | < 0.001 | 37.9 (1.6) | 37.9 (1.8) | 0.361 |
| Small for Gestational Age, n (%) | 235 (12.7) | 218 (16.6) | 0.002 | 23 (12.0) | 212 (12.7) | 0.766 |
| Birth Weight (gram), mean (SD) | 3072 (502) | 3069 (527) | 0.908 | 3099 (503) | 3068 (502) | 0.417 |
| Low Birth Weight (< 2500 g), n (%) | 204 (10.8) | 148 (10.7) | 0.947 | 24 (12.2) | 180 (10.6) | 0.510 |
| Stunted Growth, (n%) |  |  |  |  |  |  |
| - Early Childhood | 223 (22.3) | 88 (16.7) | 0.009 | 24 (27.9) | 199 (21.8) | 0.195 |
| - Mid Childhood | 77 (6.3) | 28 (6.4) | 0.961 | 8 (7.1) | 69 (6.2) | 0.724 |
| Socio-Economic Status Score, Mean (SD) | 3.4 (1.6) | 3.9 (2.2) | < 0.001 | 3.3 (1.7) | 3.4 (1.6) | 0.378 |
| Maternal Age (years), mean (SD) | 25.7 (6.2) | 26.3 (5.9) | 0.015 | 26.0 (6.4) | 25.7 (6.2) | 0.487 |
| Maternal Education, n (%) |  |  | < 0.001 |  |  | 0.201 |
| - No Formal Education | 1024 (58.2) | 687 (58.6) |  | 107 (59.4) | 917 (58.0) |  |
| - Up to Secondary | 592 (33.6) | 301 (25.7) |  | 53 (29.4) | 539 (34.1) |  |
| - Post-Secondary Education | 144 (8.2) | 184 (15.7) |  | 20 (11.1) | 124 (7.8) |  |
| Maternal Parity, n (%) | 2.2 (1.3) | 2.3 (1.4) | 0.008 | 2.2 (1.5) | 2.2 (1.3) | 0.867 |
| Mother Ever Breastfed the Participant, n (%) | 1775 (95.4) | 1226 (91.3) | < 0.001 | 188 (96.9) | 1587 (95.2) | 0.284 |

**Supplementary Table 2. Comparison of anthropometric measurements, blood pressure assessments and proportions of blood pressure status between males and females, stratified by data wave.**

| Sex | Year | Age (years) | Number | Weight (kg) | Height  (cm) | BMI  (kg/m2) | SBP  (mmHg) | DBP  (mmHg) | Normotensive  (n, (%)) | Prehypertensive  (n, (%)) | Hypertensive  (n, (%)) |
| --- | --- | --- | --- | --- | --- | --- | --- | --- | --- | --- | --- |
| Male | 1995 | 5.2 (0.2) | 503 | 18.3 (2.3) | 107.8 (4.4)* | 15.8 (1.4) | 108 (13) | 63 (8) | 353 (70.2) | 52 (10.3) | 98 (19.5) |
|  | 1998 | 8.2 (0.4) | 504 | 24.9 (3.7) | 125.0 (5.8)* | 15.9 (1.5) | 110 (10) | 69 (8)* | 334 (66.3) | 44 (8.7) | 126 (25.0) |
|  | 2002 | 12.7 (0.3) | 591 | 39.8 (8.7)*** | 147.6 (7.8)*** | 18.1 (3.3)*** | 106 (10) | 65 (8)*** | 490 (82.9) | 63 (10.7) | 38 (6.4) |
|  | 2003 | 13.7 (0.3) | 659 | 44.9 (10.2)*** | 154.6 (8.5)* | 18.7 (3.3)*** | 108 (11)* | 68 (9)*** | 501 (76.0) | 75 (11.4) | 83 (12.6) |
|  | 2005 | 15.6 (0.3)* | 780 | 54.2 (10.3)* | 165.9 (7.8)*** | 19.6 (3.1)*** | 116 (13)*** | 68 (10) | 466 (59.7)*** | 172 (22.1)*** | 142 (18.2)*** |
|  | 2008 | 17.9 (0.4) | 766 | 59.3 (9.8) | 170.8 (6.6)*** | 20.3 (3.0)*** | 121 (11)*** | 71 (9)* | 551 (71.9) | 96 (12.5) | 119 (15.5) |
|  | Overall | 12.5 (4.4) | 3803 | 41.4 (17.1)*** | 145.6 (22.8) | 18.3 (3.2)*** | 112 (13)*** | 68 (9)*** | 2695 (70.9) | 502 (13.2) | 606 (15.9) |
| Female | 1995 | 5.2 (0.2) | 518 | 18.1 (2.6) | 107.2 (4.7) | 15.7 (1.6) | 108 (12) | 64 (9) | 339 (65.4) | 48 (9.3) | 131 (25.3) |
|  | 1998 | 8.3 (0.4) | 518 | 24.7 (4.5) | 124.1 (5.7) | 16.0 (2.1) | 109 (11) | 70 (9) | 344 (66.4) | 51 (9.8) | 123 (23.7) |
|  | 2002 | 12.7 (0.3) | 649 | 45.0 (11.0) | 151.4 (7.0) | 19.5 (4.2) | 106 (10) | 67 (8) | 518 (79.8) | 65 (10.0) | 66 (10.2) |
|  | 2003 | 13.7 (0.2) | 724 | 50.2 (11.6) | 155.7 (6.2) | 20.7 (4.3) | 107 (10) | 70 (9) | 541 (74.7) | 91 (12.6) | 92 (12.7) |
|  | 2005 | 15.6 (0.2) | 837 | 55.9 (12.0) | 158.5 (6.2) | 22.2 (4.5) | 110 (12) | 68 (9) | 648 (77.4) | 92 (11.0) | 97 (11.6) |
|  | 2008 | 17.9 (0.4) | 815 | 58.9 (12.1) | 159.4 (6.1) | 23.2 (4.6) | 115 (10) | 72 (9) | 588 (72.1) | 97 (11.9) | 130 (16.0) |
|  | Overall | 12.6 (4.4) | 4061 | 43.4 (18.4) | 145.2 (23.0) | 19.9 (4.8) | 109 (11) | 69 (9) | 2978 (73.3) | 444 (10.9) | 639 (15.7) |

*BMI = Body Mass Index. SBP = Systolic Blood Pressure. DBP = Diastolic Blood Pressure. *p < 0.05 ; ***p < 0.001. Value in brackets is the standard deviation or valid percentage of the group.*

**Supplementary Table 3. Overall and sex-stratified incidence rate of hypertension per 1000 person-years and per data wave**

| **Data Wave** | **Overall** | **Males** | **Females** |
| --- | --- | --- | --- |
| 1995 | 224.3 (197.0 – 255.3) | 194.8 (159.8 – 237.5) | 252.9 (213.1 – 300.1) |
| 1998 | 170.6 (148.2 – 196.4) | 183.1 (150.8 – 222.3) | 158.6 (129.3 – 194.6) |
| 2002 | 57.1 (45.1 – 72.3) | 45.5 (31.0 – 66.9) | 67.4 (50.0 – 90.9) |
| 2003 | 72.1 (58.5 – 89.0) | 77.1 (57.5 – 103.2) | 67.5 (49.9 – 91.4) |
| 2005 | 108.8 (91.5 – 129.3) | 133.7 (106.9 – 167.1) | 84.9 (64.5 – 111.7) |
| 2008 | 88.1 (71.4 – 108.8) | 82.1 (60.0 – 112.4) | 93.7 (70.6 – 124.4) |
| Overall | 57.0 (53.2 – 61.1) | 57.1 (51.7 – 63.1) | 56.9 (51.7 – 62.7) |

*Values in brackets are the 95% confidence intervals.*

**Supplementary Table 4. Univariate survival analysis**

| **Variable** | **Log-Rank Test** | **HR** | **95% CI of HR** | | **Proportional Hazard Test** |
| --- | --- | --- | --- | --- | --- |
|  | **P-Value** |  | **Lower** | **Upper** | **P-Value** |
| Sex (Ref = Male) | 0.995 | 1.00 | 0.87 | 1.15 | 0.089 |
| Maternal Education | 0.570 |  |  |  | 0.914 |
| No Formal Education (Ref) |  | 1.00 |  |  |  |
| Up to Secondary |  | 0.92 | 0.79 | 1.07 |  |
| Post-Secondary |  | 0.97 | 0.74 | 1.28 |  |
| Maternal Parity Category (Ref = Only Child) | 0.014 | 1.18 | 1.02 | 1.37 | 0.183 |
| Birth Weight Less Than 2500 g (Ref = No) | 0.263 | 0.89 | 0.70 | 1.12 | 0.539 |
| Mother Ever Breastfed the Participant (Ref = No) | 0.846 | 0.97 | 0.70 | 1.34 | 0.522 |
| Small for Gestational Age (Ref = No) | 0.046 | 1.20 | 0.99 | 1.46 | 0.052 |
| Stunted Growth Early Childhood (Ref = No) | 0.494 | 1.07 | 0.86 | 1.34 | 0.189 |
| Stunted Growth Mid Childhood (Ref = No) | 0.153 | 0.78 | 0.54 | 1.13 | 0.156 |
| Socio-Economic Status Score (no units) | 0.122 | 0.96 | 0.92 | 1.01 | 0.588 |
| Maternal Age (years) | 0.204 | 1.01 | 1.00 | 1.02 | 0.364 |
| Maternal Parity (no units) | 0.003 | 1.08 | 1.02 | 1.13 | 0.133 |
| Gestational Age (weeks) | 0.157 | 1.03 | 0.99 | 1.07 | 0.557 |
| Birth Weight (kilograms) | 0.754 | 0.98 | 0.85 | 1.12 | 0.312 |
| Birth Weight Z-score (no units) | 0.774 | 0.99 | 0.93 | 1.05 | 0.587 |
| Duration of Breastfeeding (months) | 0.332 | 1.00 | 1.00 | 1.01 | 0.331 |
| Relative Height Gain Early Childhood (no units) | 0.973 | 1.00 | 0.91 | 1.09 | 0.815 |
| Relative Height Gain Mid Childhood (no units) | 0.073 | 1.08 | 0.99 | 1.18 | 0.496 |
| Relative Height Gain Adolescence (no units) | 0.624 | 0.98 | 0.89 | 1.07 | 0.181 |
| Relative Weight Gain Early Childhood (no units) | 0.021 | 1.11 | 1.02 | 1.21 | 0.225 |
| Relative Weight Gain Mid Childhood (no units) | 0.010 | 1.12 | 1.03 | 1.21 | 0.574 |
| Relative Weight Gain Adolescence (no units) | 0.006 | 1.14 | 1.04 | 1.26 | 0.024 |
| Relative Weight Gain Adolescence (no units) x ln(time) | 0.053 | 1.21 | 1.00 | 1.46 | N/A |
| BMI trajectories   - Normal weight (ref) - Late onset overweight - Early onset obese to overweight - Early onset obese to morbidity obese | 0.003 | 1.00  1.09  1.44  1.80 | 0.85  1.00  1.20 | 1.39  2.10  2.71 | 0.5130 |

*The variable “relative weight gain adolescence” violated the proportional hazard assumption and the univariate analysis is thus repeated by making this variable time dependent. HR = Hazard Ratio. CI = Confidence Interval. N/A = Not Applicable. Ref = Reference.*

| **Variable** | **Adjusted HR** | **95% CI of HR** | |  |
| --- | --- | --- | --- | --- |
|  |  | **Lower** | **Upper** | **P-Value** |
| Males |  |  |  |  |
| Relative Weight Gain Early Childhood (no units) | 1.13 | 0.99 | 1.30 | 0.075 |
| Relative Weight Gain Mid Childhood (no units) | 1.16 | 1.02 | 1.33 | 0.022 |
| Relative Weight Gain Adolescence (no units) x ln(time) | 1.38 | 1.05 | 1.82 | 0.021 |
| Females |  |  |  |  |
| Parity Category (Ref = Only Child) | 1.49 | 1.14 | 1.96 | 0.004 |
| Relative Weight Gain Mid Childhood (no units) | 1.10 | 0.97 | 1.24 | 0.124 |
| Relative Weight Gain Adolescence (no units) | 1.12 | 0.98 | 1.28 | 0.101 |

**Supplementary Table 5. Sex-stratified multivariable analysis**

*The variable “relative weight gain adolescence” violated the proportional hazard assumption for males only. This variable was made time dependent. Model adjusted for the variables shown. HR = Hazard Ratio. CI = Confidence Interval.*
